# Dissecting the role of radical cystectomy and urinary diversion in post-operative complications: an analysis using the American College of Surgeons national surgical quality improvement program database

**DOI:** 10.1590/S1677-5538.IBJU.2020.1098

**Published:** 2021-05-10

**Authors:** James Anaissie, Furkan Dursun, Christopher J. D. Wallis, Zachary Klaassen, Jennifer Taylor, Cinthya Obando-Perez, Jiaqiong Xu, Timothy Boone, Rose Khavari, Raj Satkunasivam

**Affiliations:** 1 Baylor College of Medicine Michael E. DeBakey Veterans Affairs Medical Center Department of Urology HoustonTexas USA Department of Urology, Baylor College of Medicine and Michael E. DeBakey Veterans Affairs Medical Center, Houston, Texas, USA; 2 Houston Methodist Hospital Department of Urology HoustonTX USA Department of Urology, Houston Methodist Hospital, Houston, TX, USA; 3 University of Texas Health Science Center Department of Urology San AntonioTX USA Department of Urology, University of Texas Health Science Center at San Antonio, TX, USA; 4 Vanderbilt University Department of Urology NashvilleTN USA Department of Urology Vanderbilt University, Nashville, TN, USA; 5 Augusta University Medical College of Georgia Division of Urology AugustaGeorgia USA Division of Urology, Medical College of Georgia - Augusta University, Augusta, Georgia, USA; 6 Houston Methodist Hospital Center for Outcomes Research HoustonTX USA Center for Outcomes Research, Houston Methodist Hospital, Houston, TX, USA

**Keywords:** Urinary Bladder Neoplasms, Urinary Diversion, Cystectomy

## Abstract

**Objective:**

To characterize the contribution of the extirpative and reconstructive portions of radical cystectomy (RC) to complications rates, and assess differences between urinary diversion (UD) types.

**Materials and Methods:**

We conducted a retrospective cohort study comparing patients undergoing UD alone or RC+UD for bladder cancer from 2006 to 2017 using ACS National Surgical Quality Improvement Program database. The primary outcome was major complications, while secondary outcomes included minor complications and prolonged length of stay. Propensity score matching (PSM) was utilized to assess the association between surgical procedure (UD alone or RC+UD) and outcomes, stratified by diversion type. Lastly, we examined differences in complication rates between ileal conduit (IC) vs. continent UD (CUD).

**Results:**

When comparing RC + IC and IC alone, PSM yielded 424 pairs. IC alone had a lower risk of any complication (HR 0.63, 95% CI 0.52-0.75), venous thromboembolism (HR 0.45, 95% CI 0.22-0.91) and bleeding needing transfusion (HR 0.41, 95% CI 0.32-0.52). This trend was also noted when comparing RC + CUD to CUD alone. CUD had higher risk of complications than IC, both with (56.6% vs 52.3%, p = 0.031) and without RC (47.8% vs 35.1%, p=0.062), and a higher risk of infectious complications, both with (30.5% vs 22.7%, p<0.001) and without RC (34.0% vs 22.0%, p=0.032).

**Conclusions:**

RC+UD, as compared to UD alone, is associated with an increased risk of major complications, including bleeding needing transfusion and venous thromboembolism. Additionally, CUD had a higher risk of post-operative complication than IC.

## INTRODUCTION

Urothelial Carcinoma of the bladder is the sixth most common malignancy in the U.S, with approximately 20% of new diagnoses being muscle invasive. Radical cystectomy (RC) with urinary diversion (UD), usually after neoadjuvant chemotherapy (NAC), is regarded as the gold standard in the treatment of muscle invasive bladder cancer (MIBC). Unfortunately, this procedure is highly morbid, with complications occurring in up to two-thirds of patients within 90 days (1). While most of these are minor, up to 20% of patients will experience a major complication, with mortality approaching 10% (2, 3).

It has been estimated that up to 60% of complications after RC are secondary to UD, yet this literature is vague and based on classification as “conduit-related complications”, which is highly subjective and at times very difficult to distinguish from complications attributable to RC (4, 5). Less commonly, UD (without RC) is performed for non-malignant etiologies, for example end stage neurogenic bladder and severe radiation cystitis. Studies have shown that UD without RC remains associated with high rates of post-operative morbidity. We hypothesize that RC significantly contributes to post-operative morbidity and mortality during RC+UD (6). We sought to characterize the additive risk RC confers in addition to UD with respect to post-operative morbidity/mortality using a contemporary dataset. To do so, we utilized the American College of Surgeons (ACS) National Surgical Quality Improvement Program (NSQIP) database, that has been shown to perform better than administrative databases or institutional series in capturing intra-operative and post-operative complications (7). Secondarily, we sought to compare the complication rates of ileal conduit and continent diversion in patients receiving those alone or following RC.

## MATERIALS AND METHODS

### 

#### Study Subjects

We utilized the participant use files of the ACS NSQIP to identify patients undergoing surgical UD, with or without concomitant RC. ACS NSQIP is a HIPAA-compliant database which documents more than 300 variables of perioperative conditions from over 600 participating institutions to measure and improve surgical quality care, for up to 30 days after the date of the procedure. Patients >18 years of age who received a surgical UD (with or without concomitant RC) were included. UD without RC included patients with ileal conduit UD (IC) (Common Procedural Terminology (CPT) code 50820) and continent UD (CUD) (CPT code 50825). UD with concomitant RC for bladder cancer (post-operative diagnosis of bladder cancer with ICD-9 code 188.x) included patients with IC, with and without lymph node dissection (CPT code 51590 and 51595, respectively), and CUD (CPT code 51596). Patients with ASA >4 and missing information during the studied post-operative period were excluded. The NSQIP database have been de-identified and this study was exempt from institutional review board approval.

#### Covariates

Relevant demographic and clinical covariates included age, sex, race, body mass index (BMI), American Society of Anesthesiologists (ASA) physical status class, history of cardiac or neurologic disease, history of chronic obstructive pulmonary disease, diabetes (requiring oral agent or insulin), end-stage renal disease requiring dialysis, current smoking status, use of pre-operative chemotherapy or radiotherapy (within 90 days of surgery), chronic steroid use, functional status prior to surgery, and total operative time. BMI was categorized in keeping with the World Health Organization stratification [<18.5, 18.5-25, 25-30, >30kg/m^2^].

#### Outcomes

Our primary outcome was major post-operative complications, including mortality, reoperation, cardiac event (myocardial infarction or cardiac arrest requiring cardiopulmonary resuscitation) or neurologic event (stroke, cerebrovascular accident or peripheral nerve injury) (8). Secondary outcomes were rates of all complications, including pulmonary complications (re-intubation or prolonged ventilation), infectious complications (surgical site infections, pneumonia, urinary tract infection or sepsis), venous thromboembolism (deep vein thrombosis or pulmonary embolism), bleeding requiring transfusion, and prolonged length of stay, comprising hospital stays greater than the median in this cohort (7 days from the date of surgery).

### Statistical Analysis

Data are presented as mean and standard deviation for continuous variables and number (percentage) for categorical variables. Propensity score matching (PSM) using the nearest neighbor algorithm was used to balance differences between demographic and clinical characteristics of patients that underwent RC+UD versus UD alone, stratified by diversion type. The propensity score was calculated from a multivariable logistic regression model utilizing all aforementioned covariates. Standardized differences (SD) were used to compare baseline characteristics of two groups, with differences less than or equal to 0.1 (10%) considered an acceptable balance (9). We assessed the likelihood of complications after propensity score matching by logistic regression. The Cox proportional hazards models were constructed to examine the associations of undergoing UD alone (compared with RC+UD) and complications. In the case of standardized differences >0.1 after PSM, the Cox proportional hazards models were adjusted for these risk factors. Proportional-hazards assumption was checked using Schoenfeld residuals. There was no violation of this assumption for any of the outcomes examined. A prior planned subgroup analyses comparing urinary diversion type used similar methodology. All analyses were performed with STATA version 16 (StataCorp. 2019. Stata Statistical Software: Release 16. College Station, TX: StataCorp LLC). Statistical significance was defined as two-tailed p <0.05 for all tests.

## RESULTS

### 

#### Comparison of Urinary Diversion Alone with Radical Cystectomy and Urinary Diversion Baseline characteristics

**Supplemental Figure-1:**
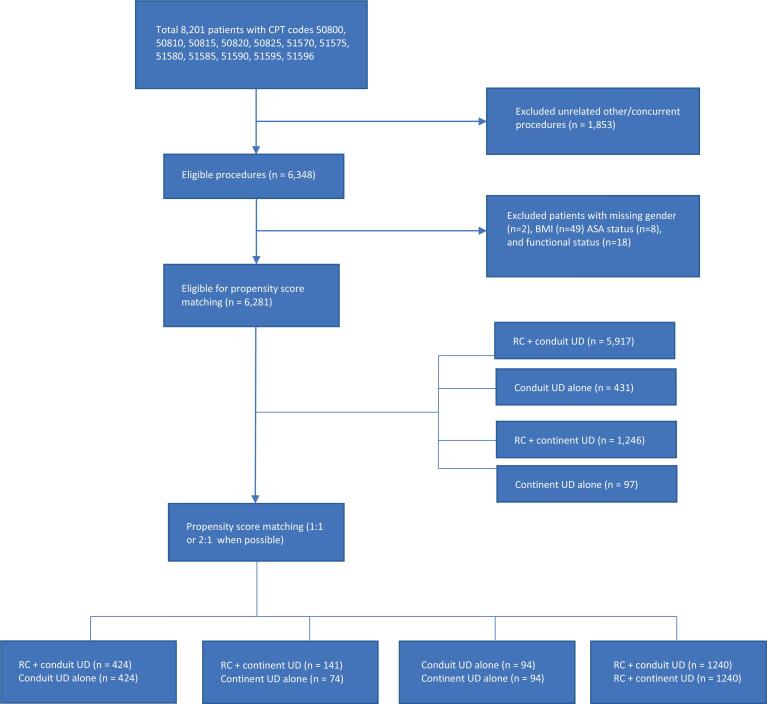
Flowchart detailing patient selection and reasons for exclusion.

We identified 7.691 patients that underwent UD who met all inclusion criteria. Of these patients, 6.348 received IC and 1.343 received CUD, with or without concomitant RC. PSM was used to match 424 patients undergoing RC+IC to 424 patients receiving IC alone. All relevant clinical and demographic variables were well balanced, with SD <0.1 (Table-1). In addition, we matched 141 patients undergoing RC+CUD to 74 undergoing CUD alone [Supplemental Figure-1]. Owing to lower numbers of patients receiving CUD, PSM was sub-optimal with notable differences in the matched cohort (Table-2). Patients who received RC+CUD were less likely to be Caucasian (84% vs. 88%, SD=0.13), but more likely to have a neurologic history (0.7% vs. 0%, SD=0.12) and required hemodialysis (1.4% vs. 0%, SD=0.17). Patients with RC+CUD were also more likely to have a longer total operative time (373±112 minutes vs. 347±116 minutes, SD=0.22).

**Table 1:** Baseline characteristics of patients before and after propensity-score matching for RC + IC vs IC alone.

		Before propensity-score matching			After propensity-score matching		
		RC+ IC	IC alone	Standardized differences	RC+ IC	IC alone	Standardized differences
Sample size (n)		5917	431		424	424	
Age, year		69.8±10.0	64.1±13.8	0.47	65.6±13.2	64.2±13.7	0.10
**Sex**				**0.59**			**-0.01**
	Male	4808 (81.3)	236 (54.8)		226 (53.3)	229 (54.0)	
	Female	1107 (18.7)	195 (45.2)		198 (46.7)	195 (46.0)	
	Missing	2 (0.03)	0 (0)		0	0	
**Race**				**0.04**			**0.03**
	Caucasian	4783 (80.8)	349 (81.0)		335 (79.0)	343 (80.9)	
	African American	272 (4.6)	32 (7.4)		39 (9.2)	32 (7.5)	
	Other/Unknown	862 (14.6)	50 (11.6)		50 (11.8)	49 (11.6)	
BMI		28.6±5.9	28.8±6.6	-0.04	28.7±7.1	28.8±6.6	-0.01
**ASA category**				**-0.02**			**0.05**
	1-2	1308 (22.1)	92 (21.3)		84 (19.8)	92 (21.7)	
	3-4	4601 (77.9)	339 (78.7)		340 (80.2)	332 (78.3)	
Cardiac history		190 (3.2)	13 (3.0)	0.01	14 (3.3)	11 (2.6)	0.04
Neurologic history		45 (0.8)	28 (6.5)	-0.31	20 (4.7)	27 (6.4)	-0.07
History of COPD		519 (8.8)	29 (6.7)	0.08	33 (7.8)	29 (6.8)	0.04
Diabetes		1285 (21.7)	92 (21.4)	0.01	92 (21.7)	88 (20.7)	0.02
Dialysis		17 (0.3)	5 (1.2)	-0.10	8 (1.9)	5 (1.2)	0.06
Active smoking		1333 (22.5)	79 (18.3)	0.10	73 (17.2)	77 (18.2)	-0.02
Pre-operative chemotherapy		79 (1.3)	4 (0.9)	0.30	1 (0.2)	4 (0.9)	0.05
Pre-operative radiotherapy		4 (0.07)	1 (0.2)	0.34	3 (0.7)	1 (0.2)	0.03
Chronic steroid use		230 (3.9)	15 (3.5)	0.02	18 (4.2)	15 (3.5)	0.04
**Functional status**				**0.46**			0.05
	Independent	5773 (97.6)	368 (85.4)		369 (87.0)	362 (85.4)	
	Partially or totally dependent	126 (2.1)	63 (14.6)		55 (13.0)	62 (14.6)	
	Unknown	18 (0.3)	0 (0)		0 (0)	0 (0)	
**Total operation time (minutes)**		**339±118**	**328±138**	**0.08**	**334±119**	**328±138**	**0.04**

**Table 2:** Baseline characteristics of patients before and after propensity-score matching for RC + CUD vs CUD alone.

		Before propensity-score matching			After propensity-score matching		
		RC + CUD	CUD alone	Standardized differences	RC + CUD	CUD alone	Standardized differences
Sample size (n)		1246	97		141	74	
Age, year		62.2 ±9.5	58.7±14.8	0.28	61.7±9.6	61.5±13.7	0.02
**Sex**				0.76			0.05
	Male	1082 (86.8)	52 (53.6)		93 (66.0)	47 (63.5)	
	Female	164 (13.2)	44 (45.4)		48 (34.0)	27 (63.5)	
	Missing	0 (0)	1 (1.0)		0	0	
**Race**				0.21			0.13
	Caucasian	1038 (83.3)	86 (88.7)		118 (83.7)	65 (87.8)	
	African American	41 (3.3)	5 (5.1)		8 (5.7)	4 (5.4)	
	Other/Unknown	167 (13.4)	6 (6.2)		15 (10.6)	5 (6.8)	
BMI		28.7±5.2	27.4±6.9	0.20	27.1±4.3	27.4±6.3	-0.05
**ASA category**				0.06			-0.09
	1 - 2	437 (35.1)	37 (38.1)		58 (41.1)	27 (36.5)	
	3 - 4	807 (64.9)	60 (61.9)		83 (58.9)	47 (63.5)	
Cardiac history		25 (2.0)	2 (2.1)	-0.004	4 (2.8)	2 (2.7)	0.01
Neurologic history		4 (0.3)	4 (4.1)	-0.26	1 (0.7)	0 (0)	0.12
History of COPD		55 (4.4)	6 (6.2)	-0.08	8 (5.7)	5 (6.8)	-0.04
Diabetes		181 (14.5)	12 (12.4)	0.06	15 (10.6)	10 (13.5)	-0.09
Dialysis		3 (0.2)	3 (3.1)	-0.22	2 (1.4)	0 (0)	0.17
Active smoking		355 (28.5)	25 (25.8)	0.06	45 (31.9)	22 (29.7)	0.05
Pre-operative chemotherapy		42 (3.4)	2 (2.1)	0.15	2 (1.4)	1 (1.3)	-0.04
Pre-operative radiotherapy		0 (0)	1 (1.0)	0.23	0 (0)	0 (0)	0
Chronic steroid use		25 (2.0)	0 (0)	0.20	0 (0)	0 (0)	0
**Functional status**				0.49			-0.01
	Independent	1236 (99.2)	83 (85.6)		135 (95.7)	71 (96.0)	
	Partially or totally dependent	9 (0.7)	12 (12.3)		6 (4.3)	3 (4.0)	
	Unknow	1 (0.1)	2 (2.1)				
**Total operation time (minutes)**		**393±131**	**325±117**	**0.55**	**373±112**	**347±116**	**0.22**

#### Bivariate analysis

Patients undergoing RC+IC were more likely to experience any post-operative complication when compared to IC alone (55.9% vs. 40.8%, p <0.001, Supplemental Table-1). They also had higher rates of mortality than those with conduit UD alone (3.1% vs. 1.2%), although this finding did not meet the conventional threshold for statistical significance (p=0.069). Patients undergoing RC+IC were also more likely to experience venous thromboembolism (5.7% vs. 2.6%, p=0.028), bleeding needing transfusion (43.2% vs. 19.6%, p <0.001), and prolonged length of stay (52.4% vs. 45.5%, p=0.046).

**Supplemental Table 1:** Rates of complications after propensity score matching – Comparison of RC + Urinary Diversion to Urinary Diversion Alone.

		RC + IC (n=424)	IC alone (n=424)	p-value	RC + CUD (n=141)	CUD alone (n=74)	p-value
**Major complication (n, %)**		30 (7.1)	30 (7.1)	1.0	16 (11.3)	9 (12.2)	0.825
Mortality (n, %)		13 (3.1)	5 (1.2)	0.069	4 (2.8)	0 (0)	0.290
Reoperation (n, %)		16 (3.8)	23 (5.4)	0.265	9 (6.4)	9 (12.2)	0.154
Cardiac complication (n, %)		7 (1.6)	3 (0.7)	0.220	3 (2.1)	0 (0)	0.383
Neurologic complication (n, %)		3 (0.7)	2 (0.5)	0.657	2 (1.4)	0 (0)	0.528
Pulmonary complication (n, %)		21 (4.9)	15 (3.5)	0.320	4 (2.8)	2 (2.7)	1.0
**Infectious complication (n, %)**		100 (23.6)	98 (23.1)	0.861	43 (30.5)	24 (32.4)	0.912
	Sepsis (n, %)	44 (10.4)	32 (7.5)	0.154	18 (12.8)	9 (12.2)	1.0
	Pneumonia (n, %)	15 (3.5)	15 (3.5)	1.0	2 (1.4)	1 (1.3)	1.0
	Urinary tract infection (n, %)	42 (9.9)	36 (8.5)	0.474	17 (12.1)	9 (12.2)	0.687
	Surgical site infection (SSI) (n, %)	61 (14.4)	46 (10.8)	0.114	26 (18.4)	15 (20.3)	0.739
	Organ space SSI (n, %)	24 (5.7)	23 (5.4)	0.882	14 (9.9)	5 (6.8)	0.382
	Deep incisional SSI (n, %)	4 (0.9)	4 (0.9)	1.0	3 (2.1)	3 (4.0)	0.396
	Superficial SSI (n, %)	34 (8.0)	21 (4.9)	0.061	10 (7.1)	7 (9.5)	0.539
**Venous thromboembolism (n, %)**		24 (5.7)	11 (2.6)	0.028	8 (5.7)	1 (1.3)	0.191
	Deep vein thrombosis (n, %)	19 (4.5)	8 (1.9)	0.056	7 (5.0)	0 (0)	0.149
	Pulmonary embolism (n, %)	9 (2.1)	3 (0.7)	0.080	3 (2.1)	1 (1.3)	0.725
Bleeding needing transfusion (n, %)		183 (43.2)	83 (19.6)	<0.001	50 (35.5)	12 (16.2)	0.006
Prolonged length of stay (n, %)		222 (52.4)	193 (45.5)	0.046	67 (47.5)	30 (40.5)	0.339
Any above complication (n, %)		237 (55.9)	173 (40.8)	<0.001	85 (60.3)	35 (47.3)	0.052

Pulmonary complication included “On Ventilator greater than 48 Hours" or Unplanned Intubation”.

p-value was obtained from conditional logistic model.

Similarly, patients receiving RC+CUD were more likely to experience any post-operative complication (60.3% vs. 47.3%, p=0.052) than CUD alone. In addition, RC+CUD as compared to CUD was associated with bleeding needing transfusion (35.5% vs. 16.2%, p=0.006) (Supplemental Table-1).

#### Regression analyses

Patients who underwent IC alone were less likely than RC+IC to have any complication (HR 0.63, 95% CI 0.52-0.75, p <0.001), venous thromboembolism (HR 0.45, 95% CI 0.22-0.91, p=0.027), and bleeding needing transfusion (HR 0.41, 95% CI 0.32-0.52, p <0.001) [Supplemental Table-2]. Patients with CUD alone were less likely than RC+CUD to experience a post-operative complication (HR 0.68, 95% CI 0.47-0.96, p=0.031) and bleeding needing transfusion (HR 0.44, 95% CI 0.23-0.84, p=0.013), as seen in Supplemental Table-2. After controlling for race, neurologic history, hemodialysis, and operative time, which were unbalanced after PSM, the risk of experiencing a post-operative complication (HR 0.71, 95% CI 0.49-1.02, p=0.067) and bleeding needing transfusion (HR 0.48, 95% CI 0.25-0.92, p=0.026) remained significant for patient with CUD alone compared with those with RC+CUD.

**Supplemental Table 2:** Association of RC with complications compared to urinary diversion alone.

	IC alone vs IC + RC			CUD alone vs RC + CUD		
	Hazards ratio	95% confidence interval	p-value	Hazards ratio	95% confidence interval	p-value
Major complications	0.98	0.59 – 1.65	0.952	1.02	0.47 – 2.20	0.956
Any complication	0.63	0.52-0.75	<0.001	0.68	0.47-0.96	0.031
Pulmonary complications	0.71	0.36 – 1.40	0.324	0.96	0.17 – 5.38	0.960
Infectious complications	0.98	0.76 – 1.27	0.913	1.10	0.68 – 1.77	0.701
Venous thromboembolism	0.45	0.22 – 0.91	0.027	0.23	0.03 – 1.89	0.173
Bleeding needing transfusion	0.41	0.32 – 0.52	<0.001	0.44	0.23 – 0.84	0.013

Hazards ratio and 95% CI was obtained from Cox proportional models with clustering on the pairs from propensity score matching. Proportional-hazards assumption was checked using Schoenfeld residuals and there was no violation for any of the outcomes.

#### Comparison of Ileal Conduit (IC) and Continent Urinary Diversion (CUD) Baseline characteristics

In order to compare the association of complications with urinary diversion complexity, we used PSM to match patients receiving IC vs. CUD, either following RC (PSM: 1.243 to 1.243) or in circumstances where UD was performed alone (PSM: 94 to 94). While PSM for IC vs. CUD with RC (Supplemental Table-3) was well balanced, PSM for IC vs. CUD alone (Supplemental Table-4) was again limited by low number of patients, leading to notable differences such that patients with CUD alone were more likely to be male and have a higher BMI, and less likely to have an ASA score >2 or a cardiac history than IC alone.

**Supplemental Table 3:** Baseline characteristics of patients before and after propensity-score matching for IC alone vs CUD alone.

		Before propensity-score matching			After propensity-score matching		
		IC alone	CUD alone	Standardized differences	IC alone	CUD alone	Standardized differences
Sample size (n)		431	97		94	94	
Age, year		64.1 ±13.8	58.7±14.8	0.38	58.1±14.0	58.6±14.9	-0.03
**Sex**				**0.01**			**-0.13**
	Male	236 (54.8)	52 (53.6)		44 (46.8)	50 (53.2)	
	Female	195 (45.2)	44 (45.4)		50 (53.2)	44 (46.8)	
	Missing	0 (0)	1 (1.0)		0	0	
**Race**				**0.22**			**0.04**
	Caucasian	349 (81.0)	86 (88.7)		82 (87.2)	83 (88.3)	
	African American	32 (7.4)	5 (5.2)		5 (5.3)	5 (5.3)	
	Other/Unknown	50 (11.6)	6 (6.2)		7 (7.5)	6 (6.4)	
BMI		28.3±7.8	27.4±6.9	0.12	26.3±7.5	27.3±6.8	-0.13
**ASA category**				**0.37**			**0.13**
	1 - 2	92 (21.4)	37 (38.1)		30 (31.9)	36 (38.3)	
	3 - 4	339 (78.6)	60 (61.9)		64 (68.1)	58 (61.7)	
Cardiac history		13 (3.0)	2 (2.1)	0.06	5 (5.3)	2 (2.1)	0.17
Neurologic history		28 (6.5)	4 (4.1)	0.11	4 (4.3)	4 (4.3)	0
History of COPD		29 (6.7)	6 (6.2)	0.02	5 (5.3)	6 (6.4)	-0.04
Diabetes		92 (21.4)	12 (12.4)	0.24	13 (13.8)	11 (11.7)	0.06
Dialysis		5 (1.2)	3 (3.1)	-0.13	4 (4.3)	3 (3.2)	0.06
Active smoking		79 (18.3)	25 (25.8)	-0.18	27 (28.7)	24 (25.5)	0.07
Pre-operative chemotherapy		4 (0.9)	2 (2.1)	-0.01	0 (0)	2 (2.1)	-0.04
Pre-operative radiotherapy		1 (0.2)	1 (1.0)	-0.02	0 (0)	1 (1.1)	-0.07
Chronic steroid use		15 (3.5)	0 (0)	0.27	0 (0)	0 (0)	0
**Functional status**				**-0.06**			0
	Independent	368 (85.4)	83 (85.5)		82 (87.2)	82 (87.2)	
	Partially or totally dep endent	63 (14.6)	12 (12.4)		12 (12.8)	12 (12.8)	
	Unknown	0 (0)	2 (2.1)		0 (0)	0 (0)	
**Total operation time (minutes)**		**328±138**	**325±118**	**0.03**	**326±142**	**324±119**	**0.01**

**Supplemental Table 4:** Baseline characteristics of patients before and after propensity-score matching for RC + IC vs RC+CUD.

		Before propensity-score matching			After propensity-score matching		
		RC+IC	RC+CUD	Standardized differences	RC+ IC	RC+CUD	Standardized differences
Sample size (n)		5917	1246		1240	1240	
Age, year		69.8 ±10.0	62.2 ±9.5	0.78	61.9±11.0	62.3±9.5	-0.03
**Sex**				**-0.15**			**0.01**
	Male	4808 (81.3)	1082 (86.8)		1082 (87.3)	1077 (86.9)	
	Female	1107 (18.7)	164 (13.2)		158 (12.7)	163 (13.1)	
	Missing	2 (0.03)	0 (0)		0	0	
**Race**				**0.05**			**0**
	Caucasian	4783 (80.8)	1038 (83.3)		1033 (83.3)	1034 (83.4)	
	African American	272 (4.6)	41 (3.3)		43 (3.5)	41 (3.3)	
	Other/Unknown	862 (14.6)	167 (13.4)		164 (13.2)	165 (13.3)	
BMI		28.6±5.9	28.7±5.2	-0.02	28.7±6.1	28.7±5.2	0.01
**ASA category**				**0.29**			**0**
	1 - 2	1308 (22.1)	437 (35.1)		435 (35.1)	436 (35.2)	
	3 - 4	4601 (77.9)	807 (64.9)		805 (64.9)	804 (64.8)	
Cardiac history		190 (3.2)	25 (2.0)	0.08	31 (2.5)	25 (2.0)	0.03
Neurologic history		45 (0.8)	4 (0.3)	0.06	4 (0.3)	4 (0.3)	0
History of COPD		519 (8.8)	55 (4.4)	0.18	65 (5.2)	54 (4.4)	0.04
Diabetes		1285 (21.7)	181 (14.5)	0.19	193 (15.6)	180 (14.5)	0.03
Dialysis		17 (0.3)	3 (0.2)	0.01	4 (0.3)	3 (0.2)	0.02
Active smoking		1333 (22.5)	355 (28.5)	-0.14	375 (30.2)	354 (28.6)	0.04
Pre-operative chemotherapy		79 (1.3)	42 (3.4)	0.12	34 (2.7)	42 (3.4)	0.01
Pre-operative radiotherapy		4 (0.07)	0 (0)	0.09	0 (0)	0 (0)	0
Chronic steroid use		230 (3.9)	25 (2.0)	0.11	22 (1.8)	25 (2.0)	-0.02
**Functional status**				**-0.12**			**-0.05**
	Independent	5773 (97.6)	1236 (99.2)		1225 (98.8)	1231 (99.3)	
	Partially or totally dependent	126 (2.1)	9 (0.7)		15 (1.2)	9 (0.7)	
	Unknown	18 (0.3)	1 (0.1)		0 (0)	0 (0)	
**Total operation time (minutes)**		**339±118**	**393±131**	**-0.44**	**393±135**	**393±130**	**0**

#### Bivariate analysis

We compared CUD to IC to determine differences in complications as a function of diversion complexity in the setting of diversion alone or following RC (Supplemental Table-5). When performed without RC, CUD had a significantly higher rate of having an infectious complication than IC alone (34.0% vs. 22.2%, p=0.032) and a higher rate of having any complication, although this finding was not statistically significant (47.8% vs. 35.1%, p=0.062). A similar finding was observed for rates of major complications (10.6% vs. 6.4%, p=0.323). This finding was also noted in patients who underwent RC+CUD, as the risk of infection was again higher in RC+CUD patients (30.5% vs. 22.7%, p <0.001). CUD had a higher risk of sepsis (12.2% vs. 8.1%, p=0.001), urinary tract infection (13.9% vs. 8.2%, p <0.001), and organ space surgical site infection (8.9% vs. 6.7%, p=0.047). Additionally, the risk of having any complication was higher for CUD (56.6%) when compared to IC (52.3%, p=0.031).

**Supplemental Table 5:** Rates of complications after propensity score matching – Comparison of IC vs. CUD, with and without RC.

		IC alone (n=94)	CUD alone (n=94)	p-value	RC + IC (n=1240)	RC + CUD (n=1240)	p-value
**Major complication (n, %)**		6 (6.4)	10 (10.6)	0.323	97 (7.8)	104 (8.4)	0.611
	Mortality (n, %)	1 (1.1)	1 (1.1)	1.0	17 (1.4)	21 (1.7)	0.517
	Reoperation (n, %)	4 (4.3)	9 (9.6)	0.177	63 (5.1)	66 (5.3)	0.783
	Cardiac complication (n, %)	0 (0)	0 (0)	1.0	25 (2.0)	26 (2.1)	0.889
	Neurologic complication (n, %)	1 (1.1)	0 (0)	0.499	6 (0.5)	6 (0.5)	1.0
Pulmonary complication (n, %)		2 (2.1)	3 (3.2)	0.657	35 (2.8)	31 (2.5)	0.623
**Infectious complication (n, %)**		19 (20.2)	32 (34.0)	0.032	282 (22.7)	378 (30.5)	<0.001
	Sepsis (n, %)	7 (7.5)	13 (13.8)	0.166	100 (8.1)	151 (12.2)	0.001
	Pneumonia (n, %)	5 (5.3)	2 (2.1)	0.273	32 (2.6)	24 (1.9)	0.278
	Urinary tract infection (n, %)	5 (5.3)	12 (12.8)	0.083	101 (8.2)	173 (13.9)	<0.001
	Surgical site infection (SSI) (n, %)	8 (8.5)	20 (21.3)	0.020	167 (13.5)	191 (15.4)	0.171
	Organ space SSI (n, %)	4 (4.3)	7 (7.5)	0.372	83 (6.7)	110 (8.9)	0.047
	Deep incisional SSI (n, %)	0 (0)	4 (4.3)	0.125	19 (1.5)	22 (1.8)	0.631
	Superficial SSI (n, %)	4 (4.3)	10 (10.6)	0.121	74 (6.0)	64 (5.2)	0.374
**Venous thromboembolism (n, %)**		2 (2.1)	1 (1.1)	0.571	63 (5.1)	70 (5.7)	0.535
	Deep vein thrombosis (n, %)	1 (1.1)	0 (0)	0.499	41 (3.3)	52 (4.2)	0.240
	Pulmonary embolism (n, %)	1 (1.1)	1 (1.1)	1.0	33 (2.7)	33 (2.7)	1.0
Bleeding needing transfusion (n, %)		14 (14.9)	15 (16.0)	0.819	451 (36.4)	414 (33.4)	0.120
Prolonged length of stay (n, %)		35 (37.2)	43 (45.7)	0.209	549 (44.3)	591 (47.7)	0.092
Any above complication (n, %)		33 (35.1)	45 (47.8)	0.062	648 (52.3)	702 (56.6)	0.031

Pulmonary complication included “On Ventilator greater than 48 Hours" or “Unplanned Intubation”.

p-value was obtained from conditional logistic model.

#### Regression analysis

When comparing diversions, CUD was more likely to have an infectious complication than IC both with RC (HR 1.40, 95% CI 1.20-1.64) and without (HR 1.75, 95% CI 1.01-3.03, p=0.047) [Supplemental Table-6], even after controlling for significant risk factors after PSM, including gender, BMI, ASA >2, and cardiac history (HR 1.76, 95% CI 1.02-3.06, p=0.044).

**Supplemental Table 6:** Hazards ratios and 95% confidence interval of IC vs CUD, with and without RC.

	CUD alone vs IC alone			CUD + RC vs IC + RC		
	Hazards ratio	95% confidence interval	p-value	Hazards ratio	95% confidence interval	p-value
Major complications	1.65	0.59 – 4.64	0.341	1.07	0.81 – 1.41	0.618
Any complication	1.40	0.93-2.11	0.109	1.08	0.98-1.19	0.119
Pulmonary complications	1.51	0.25-9.26	0.654	0.89	0.54 – 1.44	0.626
Infectious complications	1.75	1.01 – 3.03	0.047	1.40	1.20 – 1.63	<0.001
Venous thromboembolism	0.50	0.04 – 5.51	0.569	1.11	0.79 – 1.57	0.537
Bleeding needing transfusion	1.06	0.57 – 1.97	0.846	0.91	0.81 – 1.02	0.10

Hazards ratio and 95% CI was obtained from Cox proportional models with cluster on the pairs from propensity score matching. Proportional-hazards assumption was checked using Schoenfeld residuals and there was no violation for any of the outcomes.

## DISCUSSION

This current analysis of a prospectively maintained and well-annotated national dataset found that radical cystectomy and urinary diversion is associated with an increased risk of post-operative complications, bleeding needing transfusion and venous thromboembolism compared to urinary diversion alone.

Many studies estimate that the urinary diversion is what drives peri-operative complications following RC, accounting for up to 60% of all complications (3, 4). Rather than comparing outcomes for patients undergoing RC+UD compared to UD alone as we have done, others have attributed bowel, infectious, and renal related complications to the UD component of the operation, which is highly subjective (3, 4). In this analysis, RC+UD was compared to UD alone to more objectively elucidate what role RC plays in post-operative complications. We identified similar complication rates to those found in pre-existing literature (1, 10–12). Further, while the rate of having any complication was still high in UD alone (40.8% for IC, 47.3% for CUD), it was less frequent than in RC+UD (55.9% for RC+IC, 60.3% for RC+CUD). There were also specific post-operative complications such as bleeding needing transfusion and thromboembolic events which were higher in patients receiving RC+UD. Although not statistically significant, patients with RC+conduit UD were more likely to die than those undergoing conduit UD alone. This is consistent with previous work using the Nationwide Inpatient Sample (NIS), which showed that the addition of RC to UD for strictly benign etiologies led to higher rates of complications during the post-operative hospitalization (OR 1.23, 95% CI 1.03-1.48) (13).

The mechanism by which RC may add to operative complications is likely multifactorial. This includes differences in patient characteristics, increased operative time needed to perform RC, and the additive operative complexity of lymph node dissection. Patients with MIBC have significant nutritional deficiencies, and frailty and performance status are important predictors of complications (14, 15). The receipt of NAC may exacerbate these factors although a previous NSQIP analysis did not find increased rates of complications following NAC (16). On the other hand, patients receiving UD alone for benign indications such as neurogenic bladder commonly have concomitant bladder and bowel dysfunction that can delay urinary and fecal transit time and possibly lead to more urinary and GI complications and extended LOS (17). Supporting this is a previous NSQIP analysis comparing patients receiving RC for benign indications vs. malignant, which showed that at baseline, these patients were younger, had worse ASA scores, worse functional status, and more pre-operative sepsis, and led to a longer post-operative LOS (18). While we attempted to control for these factors such as operative time and receipt of chemotherapy, patients receiving RC are inherently different, and our results may be explained on the basis of residual confounding.

In terms of the higher observed rate of thromboembolic complications and bleeding with RC, it is well known that malignancy, including bladder cancer, is a potent risk factor for the development of venous thrombosis, which may be an important contributor to the difference observed in this study between patients receiving UD alone and RC+U (19, 20).

The type of urinary diversion chosen is highly dependent on surgeon, patient and disease factors. IC remains the most commonly performed UD after RC (21). While surgeon and patient preference usually determine diversion choice, our data suggests that complication rates should also be considered. In this study, CUD led to a higher complication rate than IC, regardless of presence of RC. It did not, however, demonstrate a statistically significant difference in major post-operative complications. Although the reason for increased complications is not obviously apparent, it may be due to the more complex surgical technique involved with CUD, which involves multiple sutures lines, valve mechanisms, tapered limbs, and longer operative times. Preexisting literature also shows that CUD leads to higher rate of late post-operative complications than conduit UD (22). A study comparing diversion types after robot-assisted RC has suggested that even though patients with conduit UD had more comorbidities, they were less likely to have a post-operative complication than patients receiving CUD (23). When looking specifically at NSQIP-based literature, however, the association is less clear. Some studies support that creation of CUD can independently predict rate of readmission when compared to conduit UD, while others suggest that short-term complications do not differ by diversion type, elucidating the need for further research on this topic (24).

Additionally, many techniques are being developed to improve outcomes after RC+UD and minimize complications. One such advancement is the enhanced recovery after surgery (ERAS) protocol, which is gaining widespread popularity (25). Recently, laparoscopic RC+UD is becoming increasingly utilized in hopes to minimize complications associated with open surgery, with initial results showing at least comparable outcomes to traditional RC+UD (26). An alternative to RC+UD altogether is bladder preserving therapy in patients with bladder cancer who are unfit or unwilling to undergo such a morbid procedure, and has potential for improved quality of life with similar oncologic outcomes (27).

Although novel, this study has several limitations. First, NSQIP only includes data for 30 days after the surgical procedure, but it is estimated that up to 20-60% of complications occur during this timeframe (28). Second, NSQIP lacks stage and histologic information, so while we know these patients had bladder cancer, we are unable to adjust for cystectomy in locally advanced disease. Additionally, although PSM led to well-balanced pairs when comparing RC+IC vs. IC alone, the population was too small to fully match RC+CUD to CUD alone, which is likely representative of the relative infrequency of CUD alone. Nonetheless, the utilization of PSM to better control for confounding by indication and the use of contemporary, generalizable NSQIP data allowed this study to contribute important insights into the differential contribution of radical cystectomy and urinary diversion to complications. Lastly, it is inherently difficult to generalize the outcomes to pre-existing literature, as there is much pre-existing literature demonstrating a large discordance in the consistency of data collection and urologic oncology outcome reporting (7, 29, 30). A strength of the NSQIP database however is that it collects data using standardized, clinical chart abstraction, which has been shown to be more comprehensive and reliable than administrative databases to identify complications (31).

## CONCLUSIONS

Although creation of urinary diversion has traditionally been thought to be one of the main drivers of post-operative morbidity, the addition of radical cystectomy adds significant peri-operative morbidity to the procedure. The increased 30-day complications associated with continent urinary diversions compared to ileal conduits should be considered during decision making with patients.
